# Statistical Learning Signals for Complex Visual Images in Macaque Early Visual Cortex

**DOI:** 10.3389/fnins.2020.00789

**Published:** 2020-07-31

**Authors:** Victor Vergnieux, Rufin Vogels

**Affiliations:** ^1^Laboratorium voor Neuro- en Psychofysiologie, Department of Neurosciences, KU Leuven, Leuven, Belgium; ^2^Leuven Brain Institute, KU Leuven, Leuven, Belgium

**Keywords:** statistical learning, expectation, predictions, sequence learning, visual cortex, monkey fMRI

## Abstract

Animals of several species, including primates, learn the statistical regularities of their environment. In particular, they learn the temporal regularities that occur in streams of visual images. Previous human neuroimaging studies reported discrepant effects of such statistical learning, ranging from stronger occipito-temporal activations for sequences in which image order was fixed, compared with sequences of randomly ordered images, to weaker activations for fixed-order sequences compared with sequences that violated the learned order. Several single-unit studies in macaque monkeys reported that after statistical learning of temporal regularities, inferior temporal (IT) neurons show reduced responses to learned fixed-order sequences of visual images compared with random or mispredicted sequences. However, it is unknown how other macaque brain areas respond to such temporal statistical regularities. To address this gap, we exposed rhesus monkeys (*Macaca mulatta*) to two types of sequences of complex images. The “regular” sequences consisted of a continuous stream of quartets, and within each quartet, the image order was fixed. The quartets themselves were displayed, uninterrupted, in a random order. The same monkeys were exposed to sequences of other images having a pseudorandomized order (“random” sequence). After exposure, both monkeys were scanned with functional MRI (fMRI) using a block design with three conditions: regular sequence, random sequence, and fixation-only blocks. A whole-brain analysis showed a reduced activation in mainly the occipito-temporal cortex for the regular compared to the random sequences. Marked response reductions for the regular sequence were observed in early extrastriate visual cortical areas, including area V2, despite the use of rather complex images of animals. These data suggest that statistical learning signals are already present in early visual areas of monkeys, even for complex visual images. These monkey fMRI data are in line with recent human fMRI studies that showed a reduced activation in early visual areas for predicted compared with mispredicted complex images.

## Introduction

Many species, including primates, are sensitive to spatial and temporal regularities in their environment. Behavioral studies have shown that some of these regularities can be learned [for reviews, see Krogh et al. (2012), Turk-Browne (2012), and Dehaene et al. (2015)]. For instance, the mere exposure to a set of scenes or sequences of visual stimuli is sufficient to learn their embedded statistical regularities (Fiser and Aslin, 2001, 2002). Such extraction of statistical regularities is often referred to as “statistical learning.”

Previous neuroimaging studies in humans addressed the effects of the learning of temporal regularities in sequences of visual stimuli on brain representations of those stimuli, the topic of the present study. These studies compared the activations to sequences of visual stimuli in which the order of successive stimuli within a sequence was fixed (“regular” sequence) with stimuli presented in random order (“random” sequence) or with sequences in which the stimulus order deviated from the exposed one. An earlier study (Turk-Browne et al., 2009) observed stronger activation to regular compared to random sequences of letter-like symbols in several brain regions, including the medial temporal lobe, the frontal regions, the temporal cortex, the parietal cortex, and the cuneus. Greater activations for random compared to regular sequences were not observed. Using a similar design, but with visual scenes as stimuli, a later study (Otsuka and Saiki, 2017) showed stronger activations to random compared to regular sequences in the left posterior cingulate cortex. Note that in these studies the temporal regularities during stimulus exposure were irrelevant to behavior.

In regular sequences, stimuli can be predicted from preceding ones, and thus the above functional MRI (fMRI) studies imply that predicted stimuli (in the regular sequences) show stronger activations than unpredicted stimuli (in random sequences). However, other fMRI studies, which compared activations to regular sequences with sequences in which the same images were presented at unexpected temporal positions within the sequence, showed the opposite: weaker activations to the regular compared to sequences with violations of the learned temporal order (Richter et al., 2018; Richter and de Lange, 2019). The weaker activations to expected complex, natural stimuli were observed throughout the ventral visual stream and even in V1. In both studies of Richter et al. (2018), the reduced responses to the regular sequences were observed also in regions (e.g., frontal cortex) outside the ventral stream, but these did not overlap between studies and were suggested to be task specific (Richter and de Lange, 2019). In line with the studies of Richter et al. (2018), exposure to sequences of simple stimuli with a probabilistic regularity was reported to produce less activation compared to a brief random sequence introduced within the regular sequences in (only right) V1 and prefrontal cortex (Rosenthal et al., 2016, 2018). In an implicit visual sequence learning fMRI study (Aizenstein et al., 2004), reduced activations in the visual cortex, the frontal and parietal cortices, and the striatum were observed for expected compared to unexpected colors in a regular sequence. In yet another study, brief passive exposure to unique exemplars of object categories presented in a regular sequence at different locations in the visual field showed reduced fMRI activations in multiple regions, such as the occipital cortex, the ventral temporal cortex, the frontal cortex, and the basal ganglia (Davis and Hasson, 2016). However, part of the activations might have been due to differences in eye fixation patterns between the regular and the irregular sequences, which were not monitored in this study.

Also in rodent studies of visual statistical learning, different types of differential responses were obtained in sequence learning. One study (Gavornik and Bear, 2014) found an increased electrophysiological response to predicted gratings in mouse primary visual cortex, in line with the increased BOLD activations for regular compared to random stimuli seen in higher areas in human fMRI studies (Turk-Browne et al., 2009; Otsuka and Saiki, 2017). However, a two-photon calcium imaging study reported overall weaker visual responses for predicted compared to unpredicted stimuli in the primary visual cortex (Fiser et al., 2016). Adding to the complexity, this study showed also that responses in anterior cingulate increased with stimulus predictability.

In monkeys, recordings revealed visual statistical learning effects in inferior temporal (IT) cortical spiking activity (Meyer and Olson, 2011; Meyer et al., 2014; Ramachandran et al., 2016, 2017; Schwiedrzik and Freiwald, 2017; Kaposvari et al., 2018). These studies consistently showed a reduced response to stimuli in a learned regular sequence compared to unpredicted stimuli that deviated from the learned sequence. These macaque IT results agree with the reduced BOLD activations in human ventral stream (Richter et al., 2018) using a very similar paradigm as that of Meyer and Olson. However, Kaposvari et al. (2018) employed a continuous presentation of short regular sequences of stimuli similar to a human fMRI study (Turk-Browne et al., 2009) but, contrary to that fMRI study, observed decreased responses in regular compared to random sequences. Also, Ramachandran et al. (2017) observed smaller responses in macaque IT to stimuli of learned short sequences compared to random sequences, but repetition suppression might have contributed to this effect since the stimuli of the two conditions differed also in frequency during the recordings in that study.

So far, the origin of the statistical learning effects in IT is unclear, and it is unknown which other brain regions show visual statistical learning effects in macaques. To address this gap, we employed fMRI to map the regions that show visual statistical learning signals in the monkey. We aimed to compare the activations to regular and random sequences that were presented during a continuous stream, as in a human fMRI study (Turk-Browne et al., 2009) and our earlier macaque IT recording study (Kaposvari et al., 2018). This paradigm differs from that employed in the human fMRI studies that compared activations to regular sequences and sequences in which a stimulus deviated from the learned one (Aizenstein et al., 2004; Rosenthal et al., 2016, 2018; Richter et al., 2018; Richter and de Lange, 2019). In the latter paradigm, the observed greater activations for sequences that include violations of the learned sequence could have resulted from a surprise response to the sequence violations. The presence of increased pupil dilation upon presentation of the deviant stimuli (Richter and de Lange, 2019) might be related to such a surprise response. Such potential surprise response and corresponding pupil response will also complicate the interpretation of the enhanced responses in early visual cortical areas observed in these fMRI studies. Furthermore, sequence violations can result in basal forebrain activity (Zhang et al., 2019), potentially impacting on responses in visual areas. To circumvent these complications, we compared the responses between a regular stimulus sequence and a (pseudo-)random sequence instead of a sequence with violations of a learned, regular sequence.

In the present study, we exposed two monkeys for several weeks to two sequences, a regular sequence consisting of five quartets (20 stimuli in total), in which the order of the four stimuli was fixed (transitional probabilities of 1), and a pseudorandom sequence, in which 20 other stimuli had transitional probabilities of 0.2. We controlled for differences in stimulus familiarity and stimulus repetition to exclude the possibility that the potential differences in activation between the two sequences were due to familiarity and repetition suppression. The groups of 20 stimuli of each sequence were counterbalanced across the two animals to control for stimulus-specific effects unrelated to the sequences. After exposure, we contrasted the whole-brain fMRI activations for the two sequences, aiming to reveal regions that show statistical learning-related signals.

## Materials and Methods

### Subjects and Surgery

Two female rhesus monkeys, M1 (5.5 kg; age: 6 years) and M2 (4.5 kg; age: 9 years) participated in this study. They were implanted with an MRI-compatible headpost for fixing the head during training and fMRI scanning, using standard procedures under full anesthesia and aseptic conditions. The animal care and experimental procedures complied with the regional (Flanders), European, and National Institute of Health guidelines and were approved by the Animal Ethical Committee of KU Leuven.

### Stimuli and Sequences

The stimuli consisted of 40 achromatic images of animal drawings, selected from the Snodgrass and Vanderwart image database (Snodgrass and Vanderwart, 1980)^1^. The drawings were rescaled so that their bounding box measured 4° of the visual angle in either the vertical or the horizontal dimension. Their contrast and luminance were equated using the Shine toolbox (Willenbockel et al., 2010) and presented with gamma correction of the display luminance on a gray background. A central red fixation target (size 0.2° of the visual angle) was continuously present and superimposed on the image.

**FIGURE 1:**
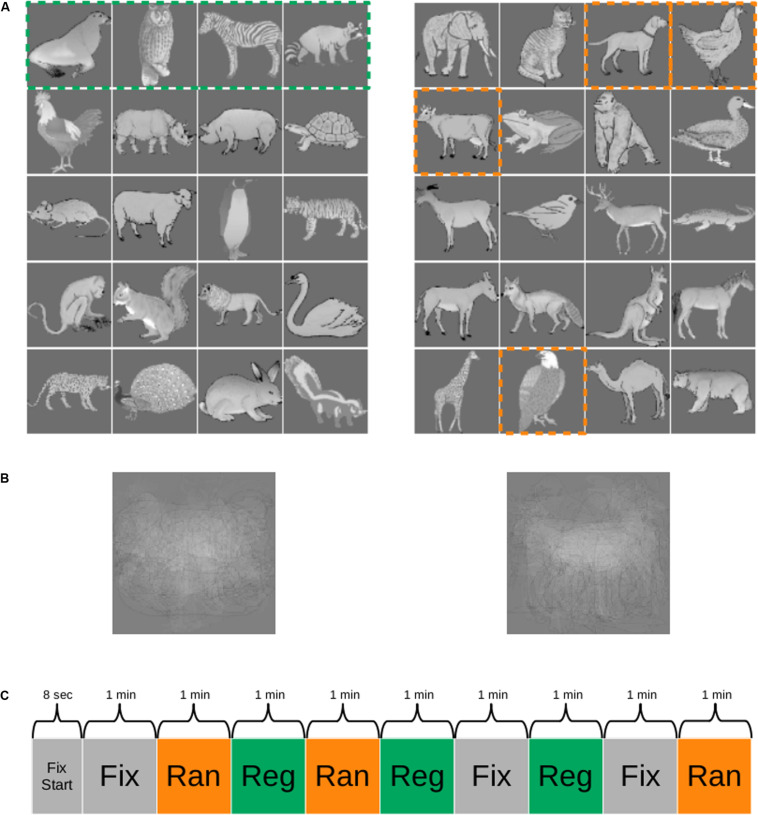
Stimuli and functional MRI (fMRI) paradigm. **(A)** One group of images (e.g., images of the left panel) was employed as the regular sequence in one monkey and as the random sequence in the other monkey and *vice versa*. The images were presented in quartets (stippled green box in the left panel), resulting in five fixed quartets (rows) for the regular sequence. The random sequences were obtained by selecting for each presented quartet at random one of the five images at each position [column in **(A)**, right panel] of the quartet. The dashed orange outlines indicate one such possible selection. In this example, the quartet would be “cow, eagle, dog, hen.” For more details, see “Materials and Methods.” **(B)** Mean of the images for each of the two groups. **(C)** Example of a run during scanning. Reg, regular sequence; Ran, random sequence; Fix, fixation only condition; Fix Start, fixation only period at the beginning of the run. The duration of each block is indicated. The order of the conditions was randomized across runs.

The 40 images were assigned to two groups of 20 images (Figure 1A). One group of 20 images was employed to create five quartets and formed the stimuli for the regular sequence. The order of the images within each quartet was fixed, but the quartets were presented in random order with the constraint that the immediate repetition of a quartet was not allowed. The other group of 20 images was divided in four sets of five stimuli each (columns in Figure 1A) and were presented in a random sequence. The stimuli were presented in a pseudorandom order in quartets so that the image at position *i* (*i* = 1–4; columns in Figure 1) of a quartet was randomly selected from the set *i* of five images. Hence, quartets were generated in which the first element would be chosen randomly from the first set of five images (first column in Figure 1A, e.g., cow), the second element chosen from the five images of the second set (second column in Figure 1A; e.g., eagle), the third element chosen from the five images of the third set (third column in Figure 1A, e.g., dog), and the last element of the quartet would be an image of the fourth set of five images (fourth column in Figure 1A, e.g., hen). Thus, the order of the images within a random quartet was pseudorandom, with transitional probabilities of 0.2 (1/5). The presentation of an image of an immediately preceding quartet was not allowed. This pseudorandomization made sure that the distribution of the time interval between repetitions of a stimulus was equal between the regular (consisting of fixed quartets) and the random sequences. The latter sequence, together with the fact that at least seven other images were present between the occurrences of the same image, served to control for repetition suppression as causing the activation differences between the two sequences.

The means of the images of each of the two groups were similar (Figure 1B). To dissociate stimulus-specific effects from statistical learning effects, we counterbalanced the stimuli across the two monkeys. Thus, the 20 stimuli of the regular sequence in one animal were employed for the random sequence in the other animal and *vice versa*.

The quartets were presented in continuous sequences without demarcation between quartets. The stimuli of a quartet were presented for 500 ms and no interstimulus interval was present between stimuli, even not between stimuli of successive quartets. Regular and random sequences were presented in alternating blocks (see below for block lengths). The first sequence of a daily session alternated between the regular and the random sequences, and we made sure that the exposure duration to the random and the regular sequence stimuli was equated within practical limits in each monkey. Monkey M1 was exposed to the regular and the random sequences in total for 1,278 and 1,290 min, respectively, a negligible 0.94% difference. For M2, the total exposure durations for the two sequences were 636 and 630 min, respectively, which is a 0.95% difference. Thus, any effect cannot be due to image familiarity since the total image exposure was highly similar for the two sequences and the small differences in exposure showed opposite trends in the two monkeys.

### Apparatus and Scanning Procedure

During the sequence exposure phase, the monkeys were seated with the head fixed in a horizontal primate fMRI chair in sphinx position in a mock MRI bore. The stimuli were displayed on a 15 × 12-in. Dell LCD monitor positioned at 57 cm from the eyes. The position and the pupil size of one eye were measured at 120 Hz with an Iscan video-based eye tracker.

During the exposure phase and scanning, a digital signal processing-based computer system, developed in-house, controlled the stimulus presentation, stimulus event timing, and juice delivery while sampling a photodiode signal corresponding to stimulus onset, vertical and horizontal eye positions, and pupil size. Time stamps of the eye parameters (1,000 Hz sampling rate), stimulus, and behavioral events were stored for offline analyses.

During scanning, the monkeys sat in a sphinx position with their heads fixed in a MR-compatible monkey chair. The chair was positioned directly in front of a screen, at a distance of approximately 59 cm. The gamma-corrected stimuli were projected (Barco 6300 LCD projector) on the translucent screen located in front of the monkey. Eye position was continuously monitored (120 Hz; Iscan) during scanning, and the animals were performing the same fixation task (see section “Task” below for description) as during the exposure phase.

The monkeys were scanned with a 3T Siemens Trio scanner following standard procedures (Vanduffel et al., 2001). Functional MRI images were acquired using a custom-made eight-channel monkey coil, a saddle-shape radial transmit-only surface coil (Ekstrom et al., 2008; Kolster et al., 2009), and a gradient-echo T2^∗^-weighted echo-planar imaging sequence (repetition time TR = 2 s, echo time TE = 13 ms, flip angle = 84°, 84 × 84 matrix, 50 slices, 1.25 × 1.25 × 1.2 mm voxel size). The slices were oriented transversally, covering the whole brain. High-resolution anatomical MRI images were acquired for each monkey in a separate session under ketamine/medetomidine anesthesia, using a single radial transmit–receive surface coil and a Magnetization-Prepared Rapid Acquisition with Gradient Echo sequence (TR = 2200 ms, TE = 3.50 ms, flip angle = 9°, 320 × 260 matrix, 208 slices, 0.4 mm isotropic voxel size). To increase the signal-to-noise ratio of the functional activations (Leite et al., 2002), the contrast agent monocrystalline iron oxide nanoparticle (MION; Molday ION; 8–11 mg/kg) was injected into the monkey femoral/saphenous vein immediately before scanning.

### Task

During exposure and scanning, the monkeys were performing a fixation task for a juice reward. The delivery and the amount of juice depended on the fixation duration (Popivanov et al., 2012) but was independent of the presentation of the quartets. Both monkeys had to fixate 1,500 ms before getting the first reward, then again for 1,500 ms for the second reward. After this, the pace accelerated and the rewards were getting larger. After fixating for 9,000 ms, they received a constant reward every 500 ms. When the monkeys were breaking the fixation, the whole schedule was reinitialized. The square fixation window had a size of approximately 2.5°. Eye tracking calibration was performed at the beginning of each session. The fixation target was present continuously and the stimulus sequence was continued during fixation breaks.

### Exposure to Sequences

The monkeys were exposed to regular and random sequences in alternating order, with each sequence block lasting for 30 min (900 quartets). Unlike during fMRI scanning, the regular sequence blocks contained 10 min of fixed sequence quartets followed by 20 min of quartets with rare deviants. A deviant was a stimulus of another quartet of the regular block. In the 20-min period, 10% of the quartets contained such a deviant stimulus at positions 2, 3, or 4 of a quartet. We added these deviant stimuli during exposure to assess statistical learning using eye movement measures (see “Results”). The monkeys were exposed in daily sessions (except during the weekend), and the session duration depended on the motivation of the animal. M1 and M2 were exposed to the sequences in 28 and 27 daily sessions, respectively. After the exposure phase, the fMRI scanning sessions started (five and nine daily scanning sessions in M1 and M2, respectively).

### fMRI Design

We employed a block design consisting of three conditions: regular sequence, random sequence, and fixation-only condition. The regular sequence consisted of only quartets without deviants. In the fixation-only condition, only the fixation target was presented on the gray background. Each run started with 8 s of fixation, followed by the three conditions in a pseudorandom order. The order of the conditions was randomized across runs, with the restriction that an immediate repetition of a condition within a run was not allowed. The three conditions were presented three times for 1 min in a run (for an example run, see Figure 1C). The total run duration was 9 min and 8 s. The number of runs in a daily scanning session depended on the motivation of the monkey.

### fMRI Data Analysis

Only runs in which the monkeys were fixating the target for at least 90% of the run duration (M1: 36 runs; M2: 17 runs) were included in the analysis. For pre-processing, we re-oriented the images and the applied slice timing correction (AFNI; NIH, United States). A non-rigid, slice-by-slice realignment within runs and affine realignment between runs within a day were performed for motion correction (Kolster et al., 2009). The mean functional images were then non-rigidly co-registered to the T1 anatomical images of the same monkey using advanced normalization tools (University of Pennsylvania, United States). In a final pre-processing step, the images were smoothed in FSL (FMRIB Software Library; University of Oxford, United Kingdom) (Smith et al., 2004) with an isotropic Gaussian kernel (full-width half-maximum: 1.5 mm).

Subsequent data analysis was performed with SPM12. All valid runs were combined in a fixed-effects model for each subject separately in their native space. They were analyzed using a general linear model (GLM) with three regressors, one for each of the three conditions, plus six additional head motion regressors (translation and rotation in three dimensions) per run. Each condition was modeled using a convolution with a Gamma function (delta = 0, tau = 8, and exponent = 0.3), modeling the MION hemodynamic response function. We computed the following GLM contrasts: random–fixation only, random–regular, and regular–random. The latter two contrasts are the main contrasts of interest, while the first contrast shows the regions activated by the visual stimuli. We employed the random condition for the first contrast because this condition is a “neutral” condition without statistical learning. The resulting *t*-maps were thresholded at *p* = 0.05, family wise error (FWE) rate, corresponding to *t* = 4.9.

To localize early visual cortical activations with respect to retinotopic maps, we transformed each monkey’s brain anatomy to the F99 common monkey space and applied the same transformation to the functional maps. We employed the probabilistic retinotopic maps in the F99 space (Zhu and Vanduffel, 2019). The probabilistic maps included the data of 13 monkeys, and the random–regular cortical activations were visualized on a cortical map of the F99 brain that included retinotopically defined areas. The retinotopic maps were thresholded by requiring that 80% of the 13 animals demonstrated the same retinotopic area for that voxel.

### Analysis of Eye Metrics

We analyzed eye metrics during the last 8 days of the pre-scanning exposure phase and during the runs that entered the fMRI analysis. The last 8 days were chosen because we expected that learning would have occurred by that time and pooling these days should yield an adequate sample size to detect learning-related differences in eye metrics. For the exposure phase, we analyzed eye movements and pupil sizes only for quartet presentations during which the monkey kept its gaze inside the fixation window. Since reliable measurements of microsaccades and pupil size required that the animal stayed inside the fixation window, those were analyzed only for the exposure phase. Since we wanted to assess the eye metrics during the entire period of the same runs that were employed to compute fMRI contrasts, we did not require that the monkey kept its gaze inside the fixation window for the eye movement analysis for the data obtained during the scanning. Data were analyzed for each monkey separately.

To analyze pupil size, we downsampled the data to 120 Hz and smoothed these with a 200-ms Hamming window. We analyzed unaborted quartet presentations that were preceded by a 2 s interval during which the eye gaze was inside the fixation window and followed by 500 ms of fixation. For each unaborted quartet presentation of the regular and the random quartets, we baseline-corrected the pupil size signal by subtracting the mean pupil size in the 500 ms period before quartet onset. Then, we averaged the baseline-corrected pupil size across unaborted quartets per condition.

To detect microsaccades in the same quartets for which we analyzed the pupil size, we employed the algorithm of Engbert and Mergenthaler (2006). The eye velocity was computed after lowpass filtering the data using a cutoff frequency of 40 Hz. Then, horizontal and vertical eye velocities (Engbert and Kliegl, 2003) were calculated as follows:

$v_{k}=\frac{s_{k+2}+s_{k+1}-s_{k}-s_{k-1}}{4}$, with *s*_*k*_ being the eye position at time *k*, after downsampling at 120 Hz. The microsaccades were detected using an elliptic threshold based on the medians of both velocities and a linear factor lambda (Engbert and Mergenthaler, 2006) of 2.36 in 2D velocity space. Moreover, only movements faster than 10°/s were taken as microsaccades. The number of microsaccades was computed for each unaborted quartet and averaged across quartets per condition. The confidence intervals of the mean were computed for each condition, and the difference between the number of microsaccades for the regular and the random conditions was tested with a Wilcoxon rank sum test. We verified that log saccade velocity increased linearly with log microsaccade amplitude (Pearson *r* of 0.82 and 0.94 in M1 and M2, respectively). For the same quartets, we computed also the standard deviation of the eye position (without lowpass filtering, but downsampled at 120 Hz) for the horizontal and the vertical dimensions.

For the eye position data sampled during fMRI, we first identified epochs during which the monkey was blinking or making very large eye movements by using an empirically determined threshold on the horizontal and the vertical eye positions. For each block, we computed the number of blinks (or large eye movements) and compared these between the random and the regular conditions with a Wilcoxon rank sum test. For the eye movement data outside the blink epochs, we computed the mean and the standard deviation of the eye position for each block of the random and the regular conditions separately for the horizontal and the vertical dimensions. Differences between conditions were tested with a Wilcoxon rank sum test.

All 95% confidence intervals of the mean were computed with bootstrapping. To do this, we employed the Matlab function “bootci” with the bias-corrected and accelerated percentile method and 10,000 samples.

## Results

We exposed two monkeys for several weeks to two sequences of visual images: one sequence that consisted of quartets in which the image order was fixed (“regular sequence”) and another sequence of different images in which the order was random (“random sequence”). Both sequences were presented during passive fixation. After exposure, we scanned the two monkeys using a block design with three conditions: regular sequence, random sequence, and fixation without image presentation (fixation only).

**FIGURE 2:**
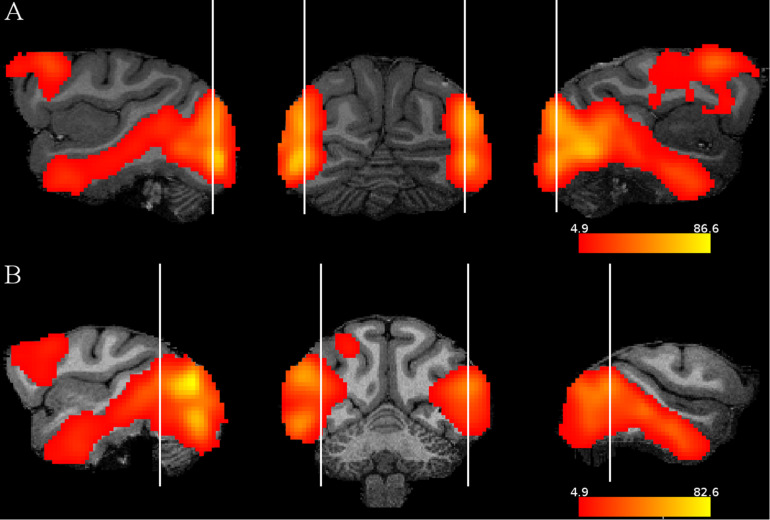
Stimulus activations: contrast random sequence – fixation only. Sagittal and coronal slices illustrating for each monkey and hemisphere the activations by the images compared to a condition in which only the fixation target was presented. The activations are presented on the MRI anatomy of each monkey. The vertical lines indicate where the slices were taken. The color scales indicate *t* values, thresholded at *p* = 0.05 family wise error rate. **(A)** M1, **(B)** M2.

**FIGURE 3:**
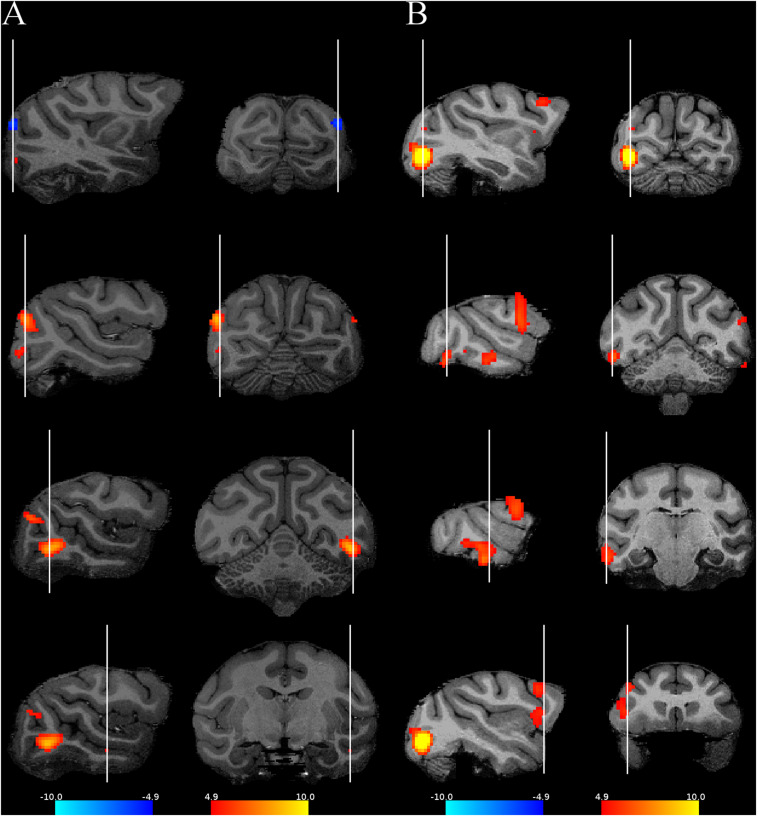
Activations when contrasting the regular and the random sequences. Four representative slices are shown for each monkey using their own anatomy. The red activations correspond to the contrast random–regular sequence, while the blue ones correspond to the opposite contrast. **(A)** M1, **(B)** M2. The same conventions as in Figure 2.

Subtracting the fixation only condition from the random image sequence showed activations by the visual images in both monkeys in mainly the occipito-temporal cortex and the frontal cortex (Figure 2), in agreement with a previous monkey fMRI study that compared activations to intact and scrambled objects (Denys et al., 2004). To isolate activations that were related to statistical learning, we computed first the contrast random–regular sequence since we expected, based on previous electrophysiological data (see “Introduction”), stronger responses to the random than the regular sequence. Examples of the activations (thresholded at *p* = 0.05 FWE rate) on anatomical MRI sections of each monkey are shown in Figure 3. This contrast showed the strongest activations in early visual cortical areas in each monkey. Activations were present also in IT, but these were rather sparse and at different regions in the two monkeys. Frontal cortical activations were present in a single hemisphere of M2.

**FIGURE 4:**
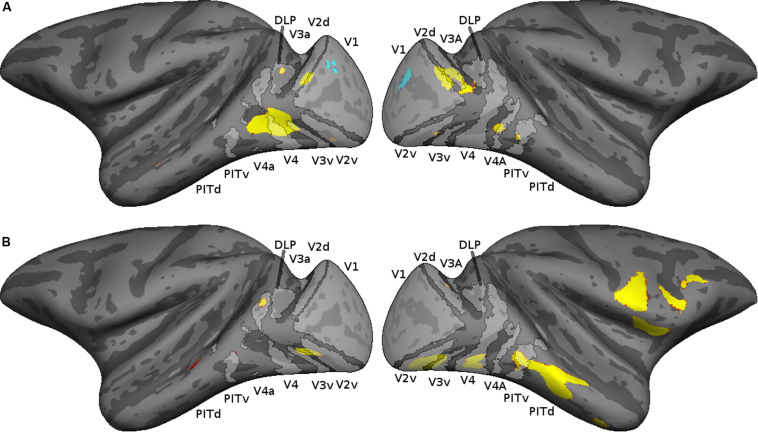
Cortical activations shown on a probabilistic retinotopic map of the inflated F99 monkey brain. The thresholded retinotopic maps are indicated in light gray, using the definitions of retinotopic areas by Zhu and Vanduffel (2019). The thresholded cortical activations (*p* < 0.05 family wise error rate) corresponding to the contrast random–regular are shown in red–yellow; those of the opposite contrast in blue. **(A)** M1, **(B)** M2.

To relate the early visual cortical activations to retinotopically defined visual areas, we mapped the cortical activations of each monkey on a probabilistic map based on the retinotopic mapping data of 13 monkeys (Zhu and Vanduffel, 2019). The cortical activations (thresholded at *p* = 0.05 FWE rate) are shown together with the probabilistic retinotopic maps on the F99 inflated brain in Figure 4. In both monkeys, stronger responses for the random compared to the regular sequences were present in areas V2, V3, and the V4 complex [DLP, V4, and V4A, as defined by Zhu and Vanduffel (2019)], with a tendency to be more prominent in the ventral visual cortex. Figure 4 also shows the activations in the IT cortex, which were present mainly in posterior IT. Anterior IT activations were present in monkey M2 but were weak and sparse in the other animal.

Subtracting the random from the regular condition showed only a weak bilateral V1 activation in M1 (Figures 3, 4) and none in M2. Hence, overall, responses were suppressed for the regular sequence images in parts of the extrastriate cortex of both animals, while enhanced responses to the regular sequences were negligible. Since the images of the random (regular) sequence of M1 were identical to the images of the regular (random) sequence in M2 and the reduced activation for the regular sequence images was present in both monkeys, the latter cannot be due to image differences between sequences. Indeed, when the activations would have resulted from differences among the images between the two sequences, one would have expected opposite activation patterns for the same subtraction in the two animals, which was not the case (Figures 3, 4).

Only in a single hemisphere of M2 did we observe activations in the frontal cortex (Figures 3, 4). A closer examination of these activations showed an overlap with the ventral premotor and the frontal operculum (insular) areas that show orofacial and gustatory responses (Maranesi et al., 2012; Kaskan et al., 2019; Sharma et al., 2019). Such responses in our setup can obviously be related to reward delivery or reward anticipation since the monkey worked for juice reward during the scanning. We compared for M2 the reward frequency and reward sizes between the two conditions in the runs that entered the contrast computation, but these were highly similar and did not differ significantly between the two conditions (median reward frequency per block: random = 62; regular = 60; rank sum test: *p* = 0.26; median reward size: random = 40; regular = 40; rank sum test: *p* = 0.34). It is possible that the animal made, for some unknown reasons, more orofacial movements in the random than in the regular condition, unrelated to the presentation of reward, but because we did not monitor the monkey’s face during scanning, we did not know. Furthermore, it is unclear why these frontal activations were unilateral since one would expect those to be bilateral for orofacial movements such as sucking. Because the frontal activations were unilateral, overlapped with orofacial activity and were present in only one monkey, we believe that these are unrelated to visual statistical learning signals.

Recent studies suggested that pupil size, being sensitive to unexpected stimulus transitions, can be used as a behavioral indicator of statistical learning (Alamia et al., 2019; Richter and de Lange, 2019). Since pupil size measures were too noisy during fMRI scanning, we analyzed the pupil sizes for the last 8 days of the pre-scanning exposure phase. In both animals, the pupil size oscillated at approximately 2 Hz, following the 2-Hz stimulus presentation rate, but we failed to find consistent differences in pupil size between the random and the regular sequences across monkeys (data not shown).

**FIGURE 5:**
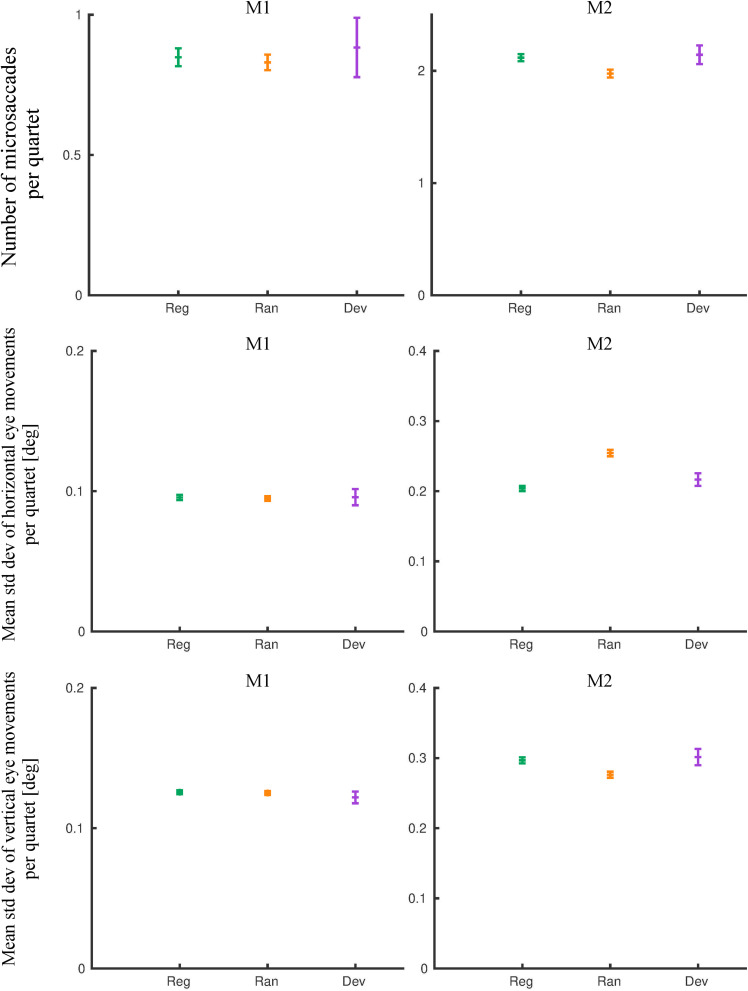
Eye movement metrics of the pre-scanning exposure phase (last eight exposure sessions). The means and 95% confidence intervals for the regular (Reg), random (Ran), and deviant (Dev) quartets are plotted for monkey M1 and M2. Std dev, standard deviation.

The mean number of microsaccades in the random and the regular quartets of the last 8 days of exposure was similar for M1 (Figure 5; rank sum test: *p* = 0.14) but differed slightly (7%) but significantly for M2 (Figure 5; rank sum test: *p* = 2.34 × 10^–8^). Similarly, the mean standard deviation of the horizontal and the vertical eye movements, computed for the same quartets, was similar for M1 for the regular and the random conditions (Figure 5; horizontal: rank sum test: *p* = 0.35; vertical: rank sum test: *p* = 0.13) but differed significantly for M2 (Figure 5; horizontal: rank sum test: *p* = 2.9 × 10^–5^; vertical: rank sum test: *p* = 0.01). However, the sign of the difference between the two conditions was opposite for the two eye movement dimensions, indicating a negligible overall difference in eye movement amplitudes between the regular and the random conditions in this monkey. An analysis of the eye movements in the quartets that included a deviant showed similar mean values to those obtained for the regular quartet in both monkeys (Figure 5).

**FIGURE 6:**
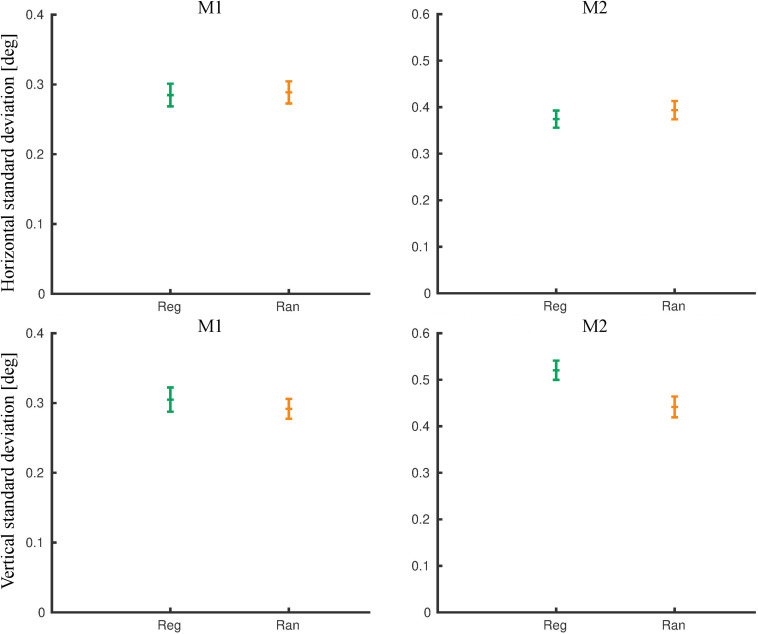
Eye movement metrics during functional MRI (fMRI) for the same runs that were employed for the fMRI analysis. The standard deviations of the eye position outside the blink epochs were computed per block for the horizontal (top) and the vertical dimensions separately. The means and 95% confidence intervals for the regular (Reg) and random (Ran) sequences are plotted for monkey M1 and M2.

During the fMRI sessions, the eye movement signal was noisier. We analyzed eye movements during the same runs that entered the fMRI contrasts. First, we removed the eye blinks. The number of blinks per block did not differ between regular and random blocks in M1 (rank sum test: *p* = 0.55) and M2 (*p* = 0.26). Then, we computed the standard deviation of the eye position outside the blink epochs (Figure 6). For M1, the mean standard deviation was similar for the horizontal (rank sum test: *p* = 0.55) and the vertical dimension (*p* = 0.42) for the two conditions. For M2, the mean standard deviation was similar for the two conditions for the horizontal dimension (*p* = 0.15) but differed significantly for the vertical dimension (*p* = 7.1 × 10^–8^), with the same trend as for the pre-scanning exposure sessions. The mean eye position did not differ significantly between the regular and the random blocks in each monkey for either dimension [rank sum test; minimum *p* = 0.16, with a maximum difference in mean position being 0.13° (vertical dimension in M2)].

The small differences in eye movements between the random and the regular conditions, seen only in one subject, and the absence of consistent pupil size differences suggest that the reduced occipito-temporal fMRI activation for the regular sequence, which was present in both monkeys, did not result from differences in eye movements, pupil size, or arousal between the two sequences.

## Discussion

Exposure to a continuous stream of images, consisting of a random sequence of quartets in which the image order was fixed, produced a reduced activation in macaque occipito-temporal cortex compared to a random sequence of images. Marked response reductions for the regular sequence were observed in early extrastriate visual cortical areas, including area V2, despite the use of rather complex images of animals. The reduced activation for the regular sequence reflected the embedded statistical regularities, being present for equally familiar images and for stimuli of the two sequences that were counterbalanced across the two subjects.

In line with the present fMRI findings, previous electrophysiological studies in macaque IT showed reduced spiking activity for regular compared to random two-image sequences (Ramachandran et al., 2017) and continuous sequences (Kaposvari et al., 2018). Because we employed complex images, we expected to see strong statistical learning effects in IT, but these were weaker than found here in early extrastriate visual areas, especially in M1. We observed, in a previous study, that the difference in spiking activity between the regular and the random sequence is mainly present for the early phase of the IT spiking response (Kaposvari et al., 2018). Unlike early visual cortical areas, IT shows strongly sustained responses, and hence the transient neural response difference between the two sequences might be proportionally small in the hemodynamic response, which reflects the complete, both early and late, sustained response.

Functional MRI studies in humans have observed pronounced reductions to regular sequences in the ventral visual stream, when compared to an unexpected, “deviant” stimulus in the sequence (Richter et al., 2018; Richter and de Lange, 2019). The reductions observed in the human ventral stream appear to be much stronger than those seen in the present study. Apart from several technical differences between studies, one potential factor explaining the difference between the human and the monkey fMRI data is that, in the human experiments, the participants were performing an active task that required attention to the images. In our study, the monkeys were performing a passive fixation task in which attention to the stimuli is uncontrolled. A recent human fMRI study showed that when attention was directed away from the images, enhanced activation to the deviant stimulus was absent (Richter and de Lange, 2019). Note however, that distracting attention away from the images by an orthogonal task (Richter and de Lange, 2019) can suppress visual responses to a larger extent than passive fixation, during which the subject can still attend the stimuli. Furthermore, single-unit IT studies have consistently observed statistical learning signals during passive fixation in monkeys (Meyer and Olson, 2011; Meyer et al., 2014; Ramachandran et al., 2016, 2017; Schwiedrzik and Freiwald, 2017; Kaposvari et al., 2018). Thus, we believe that it is unlikely that attentional factors can explain the difference between the human and the monkey fMRI findings. A potentially more likely explanation is that part of the response difference in the human fMRI study may have resulted from an additional “surprise response” to the stimulus that violated the learned sequence. Indeed we observed in a previous electrophysiological study that, in monkey IT, the spiking response to a “deviant” is stronger and more sustained than that for the random sequence [Kaposvari et al., 2018; but see Ramachandran et al. (2017)].

Two human fMRI studies that compared regular and random sequences (Turk-Browne et al., 2009; Otsuka and Saiki, 2017), as we did here, reported the opposite effect: an increased activation for the regular sequence. The reason for this discrepancy between our monkey fMRI study and the human fMRI studies is unclear, but one important factor might be the exposure duration, which was brief (i.e., during the single fMRI session) in the human studies. Although we cannot exclude it, the discrepancy between the monkey and the human fMRI studies is unlikely to reflect a species differences since our macaque results are in line with human fMRI studies that observed a reduced activation to a regular compared to a deviant stimulus sequence (Aizenstein et al., 2004; Rosenthal et al., 2016, 2018; Richter et al., 2018; Richter and de Lange, 2019). Furthermore, the latter human fMRI studies are consistent with single-unit studies in macaques that employed a highly similar paradigm (Meyer and Olson, 2011; Schwiedrzik and Freiwald, 2017).

We observed random–regular activations only in the visual occipito-temporal cortex, except for frontal activations in one hemisphere of one of the subjects. We believe that this frontal activation is artifactual, related to orofacial movements. fMRI studies in humans have reported activations related to statistical learning in non-visual areas (see “Introduction”), but these can be task-related (Richter and de Lange, 2019). Although we cannot exclude that we missed areas outside the visual cortex, the prominent activations in the visual cortex agrees with the idea that the suppression of the response to the regular sequence originates within the visual cortex, perhaps reflecting predictive coding (Friston, 2005) based on recurrent interactions between ventral areas that build object representations.

It might be surprising that statistical learning signals were present for these complex images as early as area V2. Note however, that some fMRI studies that compared expected and unexpected complex images also found activations in early visual areas, including even area V1 (Richter et al., 2018; Richter and de Lange, 2019). These human fMRI studies attributed the V1 activations to general stimulus-unspecific response modulations in arousal or to luminance changes due to pupil size differences between the unexpected deviant and the regular stimuli. Indeed pupil dilation correlated with increased V1 activation in the human fMRI study (Richter and de Lange, 2019). Pupil-related effects cannot account for the early visual activations in our study since no consistent differences in pupil size were observed between the random and the regular sequences.

Our animals were exposed to stimulus regularities for a long period. The long exposure may have led to the response changes in early visual areas for these complex images. Extensive perceptual learning of complex stimuli also produces changes in activation of early visual areas (Sigman et al., 2005). Furthermore, single-cell studies in the macaque suggested the encoding of shape category in early visual cortex (Ko and von der Heydt, 2018). Interestingly, a recent monkey single-cell study observed a reduced response to familiar compared to unfamiliar complex images in area V2 (Huang et al., 2018). It is possible that the early visual cortical modulations of the responses to complex images by image familiarity or by predictions reflect feedback from higher areas that respond to complex object features. Indeed, human fMRI studies suggested that feedback from higher areas results in the encoding of high-level stimulus features in early visual cortex (Williams et al., 2008; Fan et al., 2016; Morgan et al., 2019). This is in line with the recurrent nature of the ventral visual stream connectome (Kravitz et al., 2013), which can support the integration of predictions and feature representations at different stages of processing.

## Data Availability Statement

The raw data supporting the conclusions of this article will be made available by the authors, without undue reservation.

## Ethics Statement

The animal study was reviewed and approved by the Ethische Commissie Dierproeven KU Leuven.

## Author Contributions

VV and RV contributed to the design of the study and revised the manuscript. VV performed the experiments, conducted the analyses, and prepared the figures. RV wrote the first draft of the manuscript. Both authors contributed to the article and approved the submitted version.

## Conflict of Interest

The authors declare that the research was conducted in the absence of any commercial or financial relationships that could be construed as a potential conflict of interest.

## Footnotes

1 https://wiki.cnbc.cmu.edu/Objects
